# The IL-23/IL-22/IL-18 axis in murine *Campylobacter jejuni* infection

**DOI:** 10.1186/s13099-016-0106-4

**Published:** 2016-07-06

**Authors:** Markus M. Heimesaat, Ursula Grundmann, Marie E. Alutis, André Fischer, Ulf B. Göbel, Stefan Bereswill

**Affiliations:** Department of Microbiology and Hygiene, Charité-University Medicine Berlin, CC5, Campus Benjamin Franklin, FEM, Garystr. 5, 14195 Berlin, Germany

**Keywords:** *Campylobacter jejuni*, In vivo infection, IL-23/IL-22/IL-18 axis, IL-17A, IL-1β, Pro-inflammatory immune responses, Systemic immune responses, Translocation, Intestinal microbiota, Colonization resistance, Apoptosis

## Abstract

**Background:**

Human *Campylobacter jejuni* infections are worldwide on the rise. Information about the distinct molecular mechanisms underlying campylobacteriosis, however, are scarce. In the present study we investigated whether cytokines including IL-23, IL-22 and IL-18 sharing pivotal functions in host immunity were involved in mediating immunopathological responses upon *C. jejuni* infection.

**Results:**

To address this, conventionally colonized IL-23p19^−/−^, IL-22^−/−^ and IL-18^−/−^ mice were perorally infected with *C. jejuni* strain ATCC 43431. Respective gene-deficient, but not wildtype mice were susceptible to *C. jejuni* infection and could be readily colonized with highest pathogenic loads in the terminal ileum and colon at day 14 postinfection (p.i.). In IL-23p19^−/−^, IL-22^−/−^ and IL-18^−/−^ mice viable *C. jejuni* were detected in MLNs, but did not translocate to spleen, liver, kidney and blood in the majority of cases. Susceptible IL-22^−/−^, but neither IL-23p19^−/−^, nor IL-18^−/−^ mice harbored higher intestinal commensal *E. coli* loads when compared to resistant wildtype mice. Alike *C. jejuni*, commensal *E. coli* did not translocate from the intestinal to extra-intestinal tissue sites. Despite *C. jejuni* infection, mice lacking IL-23p19, IL-22 or IL-18 exhibited less apoptotic cells, but higher numbers of proliferating cells in their colonic epithelium as compared to wildtype mice at day 14 p.i. Less pronounced apoptosis was parallelled by lower abundance of neutrophils within the colonic mucosa and lamina propria of infected IL-23p19^−/−^ and IL-22^−/−^ as compared to wildtype control mice, whereas less distinct colonic TNF secretion could be measured in IL-22^−/−^ and IL-18^−/−^ than in wildtype mice at day 14 p.i. Notably, in infected IL-22^−/−^ mice, colonic IL-23p19 mRNA levels were lower, whereas the other way round, colonic IL-22 expression rates were lower in IL-23p19^−/−^ mice as compared to wildtype controls. Moreover, IL-18 mRNA was less distinctly expressed in large intestines of naive and infected IL-22^−/−^ mice, but not vice versa, given that IL-22 mRNA levels did not differ between in IL-18^−/−^ and wildtype mice.

**Conclusion:**

Cytokines belonging to the IL-23/IL-22/IL-18 axis mediate immunopathological responses upon murine *C. jejuni* infection in a differentially orchestrated manner. Future studies need to further unravel the underlying regulatory mechanisms orchestrating pathogenic-host interaction.

**Electronic supplementary material:**

The online version of this article (doi:10.1186/s13099-016-0106-4) contains supplementary material, which is available to authorized users.

## Background

The Gram-negative bacterium *Campylobacter jejuni* represents a major agent causing food-borne gastroenteritis in humans with rising incidences worldwide [1, 2]. In many wild and domestic animal species, *C. jejuni* is part of the commensal intestinal microbiota. Zoonotic transmission from livestock animals to humans commonly takes place via consumption of contaminated meat products or water [3–5]. *C. jejuni* infected individuals present a broad range of symptoms including mild, non-inflammatory, and watery diarrhea, but also severe, inflammatory, bloody diarrhea associated with abdominal cramps that might last for up to a few weeks, that usually resolve spontaneously [6–8]. In rare cases, however, post-infectious sequelae such as reactive arthritis and peripheral neuropathies including Guillain–Barré and Miller–Fisher syndromes might arise later on [6, 9]. Acute campylobacteriosis is characterized by histological changes of the large intestinal mucosa including apoptosis, crypt abscesses, ulcerations, whereas a pronounced influx of pro-inflammatory immune cell subsets such as lymphocytes, neutrophils, macrophages and monocytes into the intestinal mucosa and lamina propria of infected patients can be observed [7, 10]. In the past years, our understanding of the molecular mechanisms underlying campylobacteriosis was hampered by the scarcity of appropriate in vivo models. Murine models of *C. jejuni* infection exhibit some disadvantages such as sporadic pathogenic colonization, absence of overt disease and/or lack of intestinal immunopathology [6, 11]. Adult mice (beyond 2 months of age) harboring a conventional intestinal microbiota display a strong physiological colonization resistance preventing the host from pathogenic infection [11]. Colonization resistance, however, can be overcome following modulation of the conventional intestinal microbiota by several means. For instance, broad-spectrum antibiotic treatment depleting the intestinal microbiota subsequently facilitated stable *C. jejuni* colonization [12, 13]. Furthermore, conventional adult mice displaying elevated commensal *E. coli* loads in their gastrointestinal tract could be stably infected with *C. jejuni*, whereas control mice successfully expelled the pathogen within a few days postinfection (p.i.) [12–15]. Even though infected mice did not display overt symptoms of *C. jejuni* infection such as wasting or bloody diarrhea, for instance, distinct proinflammatory immune responses including a prominent influx of innate and adaptive immune cell populations into the large intestinal mucosa and lamina propria, increased colonic secretion of pro-inflammatory cytokines and higher abundances of colonic epithelial apoptotic cells could be observed postinfection, hence mimicking immunopathological key features of human campylobacteriosis [12, 13].

Our group recently showed that IL-23p19, IL-22 and IL-18 were upregulated in the colon of *C. jejuni* infected gnotobiotic (i.e. secondary abiotic) mice [16], whereas IL-22 was upregulated in *C. jejuni* infected IL-10 deficient mice [17]. IL-22 is a member of the IL-10 cytokine family and known for its antimicrobial and tissue-protective, but also pro-inflammatory properties [18, 19]. Notably, IL-22 acts literally like a double-edged sword in the intestinal tract depending on the respective compartment. Namely, in the colon, IL-22 exerts its anti-inflammatory properties [19], whereas in the small intestines, however, IL-22 acts as an pro-inflammatory mediator, given that in acute murine ileitis following peroral high dose *Toxoplasma gondii* infection, immunopathology was characterized by an IL-23p19 dependent IL-22 up-regulation leading to small intestinal necrosis [20, 21]. Whereas IL-22 further induced the expression of IL-18 mRNA in intestinal epithelial cells during *T. gondii* ileitis, IL-18 amplified IL-22 production from innate lymphoid cells (ILCs) and T helper (Th) -1 cell mediated intestinal inflammation [21]. It is, however, not known yet, whether such a mutual regulation between IL-22 and IL-18 might also apply during *C. jejuni* infection.

In the present study we therefore aimed to shed further light onto the impact of cytokines belonging to the IL-23/IL-22/IL-18 axis during *C. jejuni* infecton. To address this, we infected conventional IL-23p19^−/−^, IL-22^−/−^, IL-18^−/−^ and corresponding wildtype (WT) mice perorally with *C. jejuni* strain ATCC 43431 and investigated (1) the gastrointestinal colonization and translocation properties of *C. jejuni* as well as of commensal *E. coli* facilitating pathogenic infection, (2) the clinical outcome, (3) the subsequent apoptotic changes of the colon epithelium, (4) the abundances of distinct immune cell populations in the colonic mucosa and lamina propria, and finally (5) the large intestinal expression of inflammatory and regulatory cytokines.

## Methods

### Ethics statement

All animal experiments were conducted according to the European Guidelines for animal welfare (2010/63/EU) with approval of the commission for animal experiments headed by the “Landesamt für Gesundheit und Soziales” (LaGeSo, Berlin, registration number G0135/10). Animal welfare was monitored twice daily by assessment of clinical conditions.

### Mice and *C. jejuni* infection

Female IL-23p19^−/−^, IL-22^−/−^ and IL-18^−/−^ mice (all in C57BL/6j background) as well as age- and sex-matched C57BL/6j WT control mice, all harboring a conventional intestinal microbiota, were bred and maintained within the same specific pathogen free (SPF) unit in the Forschungseinrichtungen für Experimentelle Medizin (FEM), Charité—University Medicine Berlin. In order to confirm absence of IL-23p19, IL-22 or IL-18 gene expresion, genomic DNA was isolated and disruption of either gene confirmed by polymerase chain reaction (PCR) [20]. On 3 consecutive days (days 0, 1 and 2) mice were perorally infected with 10^9^ colony forming units (CFU) of viable *C. jejuni* strain ATCC 43431 in a volume of 0.3 mL phosphate buffered saline (PBS) by gavage as described earlier [12].

### Sampling procedures

Mice were sacrificed at day 14 p.i. by isofluran treatment (Abbott, Greifswald, Germany). Cardiac blood and tissue samples from colon, mesenteric lymph nodes (MLNs), spleen, liver and kidney were asserved under sterile conditions. Colonic ex vivo biopsies were collected in parallel for immunohistochemical, microbiological, and immunological analyses. Immunohistopathological changes were assessed in sections (5 μm) of colonic samples that were immediately fixed in 5 % formalin and embedded in paraffin.

### Immunohistochemistry

In situ immunohistochemical analysis of colonic paraffin sections was performed as described previously [16, 22, 23]. Primary antibodies against cleaved caspase-3 (Asp175, Cell Signaling, Beverly, MA, USA, 1:200), Ki67 (TEC3, Dako, Denmark, 1:100), myeloperoxidase (MPO-7, #A0398, Dako, 1:500), and F4/80 (#14-4801, clone BM8, eBioscience, San Diego, CA, USA, 1:50) were used. For each animal, the average number of positively stained cells within at least six high power fields (HPF, 0.287 mm^2^, 400× magnification) were determined microscopically by a double-blinded investigator.

### Quantitative analysis of bacterial colonization and translocation

Viable *C. jejuni* were detected in feces over time p.i. or luminal colonic samples at time of necropsy (day 14 p.i.), dissolved in sterile PBS and serial dilutions cultured on Karmali- and Columbia-agar supplemented with 5 % sheep blood (Oxoid) for 2 days at 37 °C under microaerobic conditions using CampyGen gas packs (Oxoid). To quantify bacterial translocation, ex vivo biopsies derived from MLNs, spleen, liver and kidney were homogenized in 1 mL sterile PBS, whereas cardiac blood (≈100 μL) was directly streaked onto Karmali-Agar and Columbia-Agar supplemented with 5 % sheep blood, and additionally tranferred to thioglycollate broths and cultivated accordingly. Viable *Escherichia coli* were quantitatively assessed as described earlier [24]. The respective weights of fecal or tissue samples were determined by the difference of the sample weights before and after asservation. The detection limit of viable pathogens by direct plating was ≈100 CFU per g.

### Cytokine detection in supernatants of colonic ex vivo biopsies

Colonic ex vivo biopsies were cut longitudinally, and washed in PBS. Strips of approximately 1 cm^2^ intestinal tissue were placed in 24-flat-bottom well culture plates (Nunc, Wiesbaden, Germany) containing 500 μL serum-free RPMI 1640 medium (Gibco, life technologies, Paisley, UK) supplemented with penicillin (100 U/mL) and streptomycin (100 µg/mL; PAA Laboratories). After 18 h at 37 °C, culture supernatants or serum samples were tested for TNF by the Mouse Inflammation Cytometric Bead Assay (CBA; BD Biosciences) on a BD FACSCanto II flow cytometer (BD Biosciences).

### Real-time PCR

RNA was isolated from snap frozen colonic ex vivo biopsies, reverse transcribed and analyzed as described previously [20]. Murine IL-23p19, IL-22, and IL-18 mRNA expressions were detected by real-time polymerase chain reaction (PCR) with specific primers and quantified by analysis with the light cycler data analysis software (Roche). The mRNA of the housekeeping gene for hypoxanthine-phosphoribosyltransferase (HPRT) was used as reference, the mRNA expression levels of the individual genes were normalized to the lowest measured value and expressed as fold expression (arbitrary units).

### Statistical analysis

Medians and levels of significance were determined using Mann–Whitney test (GraphPad Prism v6.05, La Jolla, CA, USA) as indicated. Two-sided probability (*P*) values <0.05 were considered significant.

## Results

### Colonization and translocation properties of *C. jejuni* in conventionally colonized mice lacking IL-23p19, IL-22 or IL-18

**Fig. 1:**
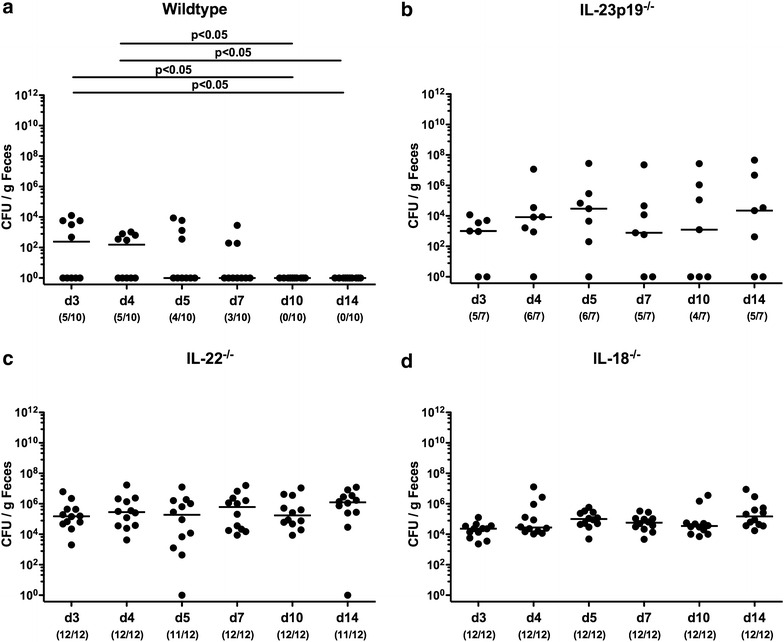
Intestinal *C. jejuni* colonization in mice lacking IL-23p19, IL-22 or IL-18. Conventional **a** wildtype, **b** IL-23p19^−/−^, **c** IL-22^−/−^ and **d** IL-18^−/−^ mice were perorally infected with *C. jejuni* strain ATCC 43431 by gavage at days 0, 1 and 2. Pathogenic loads were determined in fecal samples (*CFU* colony forming units per gram) at distinct time points (*d* day) postinfection (p.i.) as indicated by culture. Numbers of mice harboring *C. jejuni* out of the total number of analyzed animals are given in parentheses. Medians (*black bars*) and levels of significance (p values) determined by Mann–Whitney U test are indicated. Data were pooled from three independent experiments

**Fig. 2:**
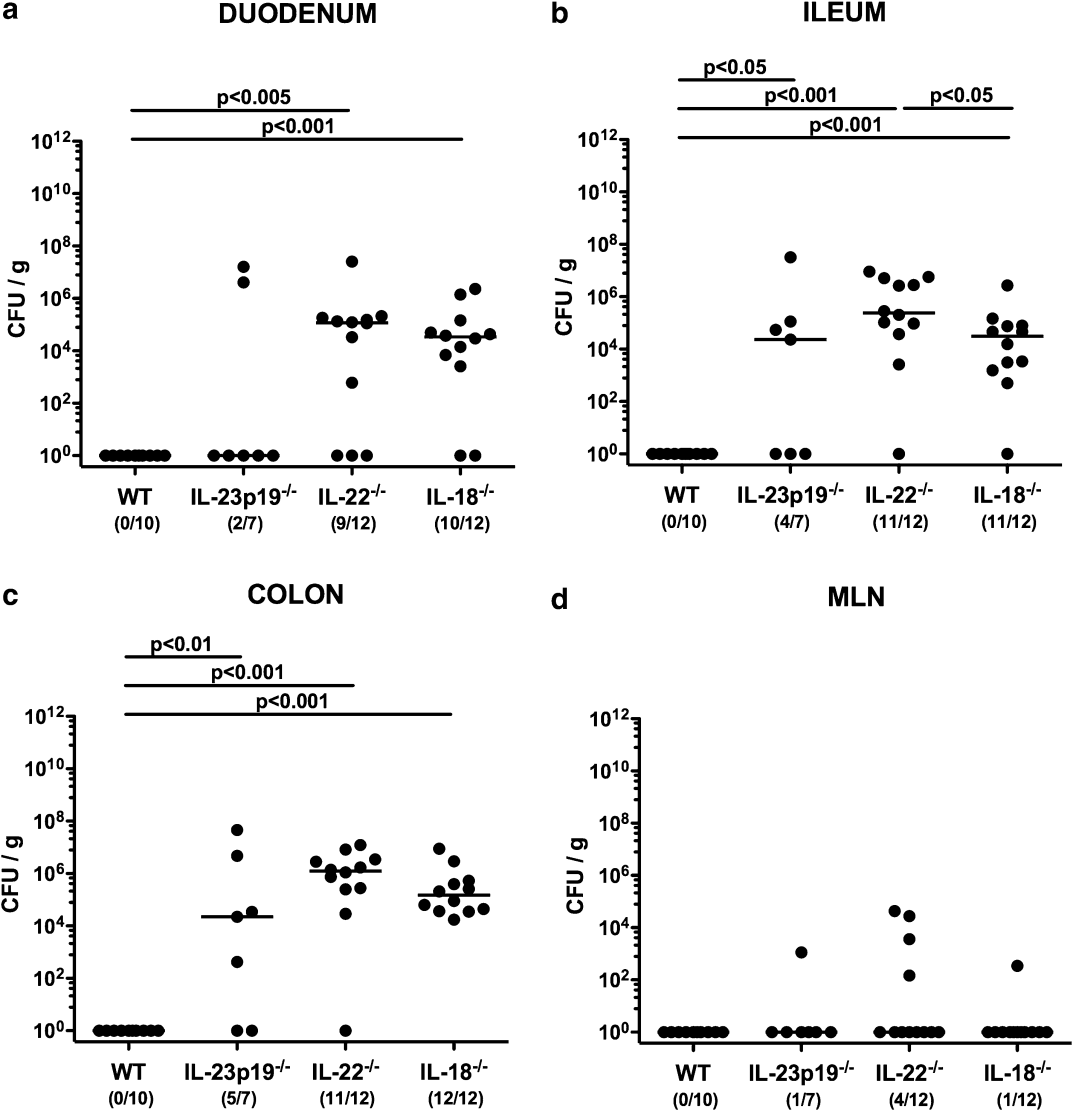
Intestinal *C. jejuni* loads in mice lacking IL-23p19, IL-22 or IL-18. Conventional wildtype (WT), IL-23p19^−/−^, IL-22^−/−^ and IL-18^−/−^ mice were perorally infected with *C. jejuni* strain ATCC 43431 by gavage at days 0, 1 and 2. Pathogenic loads (*CFU* colony forming units per gram) were determined in luminal samples derived from the **a** duodenum, **b** terminal ileum, **c** colon and homogenates of **d** mesenteric lymph nodes (MLN) at day 14 postinfection by culture. Numbers of mice harboring *C. jejuni* out of the total number of analyzed animals are given in parentheses. Medians (*black bars*) and levels of significance (p values) determined by Mann–Whitney U test are indicated. Data were pooled from three independent experiments

In the present study we investigated the role of cytokines belonging to the IL-23/IL-22/IL-18 axis within the interplay of *C. jejuni* infection, the host innate immune system, and the intestinal microbiota. To address this, adult (i.e. 3-months old) IL-23p19^−/−^, IL-22^−/−^, IL-18^−/−^ and WT mice harboring a conventional microbiota were perorally challenged with 10^9^ CFU of *C. jejuni* strain ATCC 43431 on 3 consecutive days (day 0, 1 and 2) by gavage. As early as 24 h following the latest infection (i.e. day 3 p.i.), already 50 % of WT mice had expelled the pathogen from their intestinal tract and were all culture-negative by day 10 p.i., whereas *C. jejuni* could be isolated from fecal samples at median loads of 10^4^ to 10^5^ CFU per gram in more than 70 % of IL-23p19^−/−^ and 100 % of IL-22^−/−^ and IL-18^−/−^ mice until the end of the experiment (Fig. 1). At day of necropsy (i.e. day 14 p.i.), *C. jejuni* loads assessed in luminal samples derived from the colon and ileum were higher in either gene-deficient mice as compared to WT controls (p < 0.05–0.001; Fig. 2). Moreover, IL-22^−/−^ and IL-18^−/−^ mice harbored higher pathogenic loads in the duodenum than WT mice (p < 0.005 and 0.001, respectively; Fig. 2). Interestingly, viable *C. jejuni* could be isolated from 14.3, 33.3, and 8.3 % of MLNs taken from IL-23p19^−/−^, IL-22^−/−^, and IL-18^−/−^ mice, respectively, but in none of WT control animals at day 14 p.i. (Figure 2d). Notably, irrespective of their genotype, mice were not compromized upon infection and did not exhibit symptoms of campylobacteriosis such as bloody diarrhea (not shown).

We next investigated whether *C. jejuni* were able to translocate from the intestinal tract to extra-intestinal compartments. In single cases, viable bacteria could be isolated from spleen (28.6 and 16.7 %), liver (14.3 and 16.7 %), and kidney (14.3 and 25.0 %) of IL-23p19^−/−^ and IL-22^−/−^ mice, respectively, but none of IL-18^−/−^ and WT animals by direct plating (Additional file1: Fig. S1). Notably, all blood cultures were *C. jejuni* culture-negative as assessed by direct plating plus enrichment broths (Additional file 1: Fig. S1). Hence, *C. jejuni* was able to stably colonize the intestines of virtually all IL-22^−/−^ and IL-18^−/−^, approximately 75 % of IL-23p19^−/−^, but not WT mice, with highest luminal loads within the large intestines. Only in single cases, however, viable pathogen could be detected in extra-intestinal compartments, but never in cardiac blood.

### Intestinal commensal *E. coli* loads in conventionally colonized mice lacking IL-23p19, IL-22 or IL-18

**Fig. 3:**
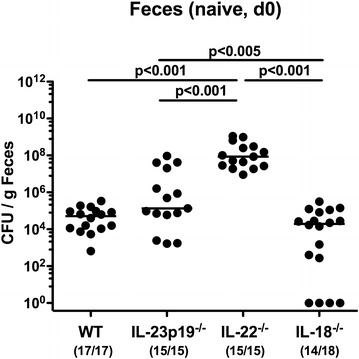
Fecal *E. coli* loads in conventional mice lacking IL-23p19, IL-22 or IL-18. Commensal *E. coli* loads (*CFU* colony forming units per gram) were quantitated in fecal samples derived from conventional wildtype (WT), IL-23p19^−/−^, IL-22^−/−^ and IL-18^−/−^ infant naive mice before *C. jejuni* strain ATCC 43431 infection (day (d) 0). Numbers of mice harboring *E. coli* out of the total number of analyzed animals are given in parentheses. Medians (*black bars*) and levels of significance (p values) determined by Mann–Whitney U test are indicated. Data were pooled from three independent experiments

**Fig. 4:**
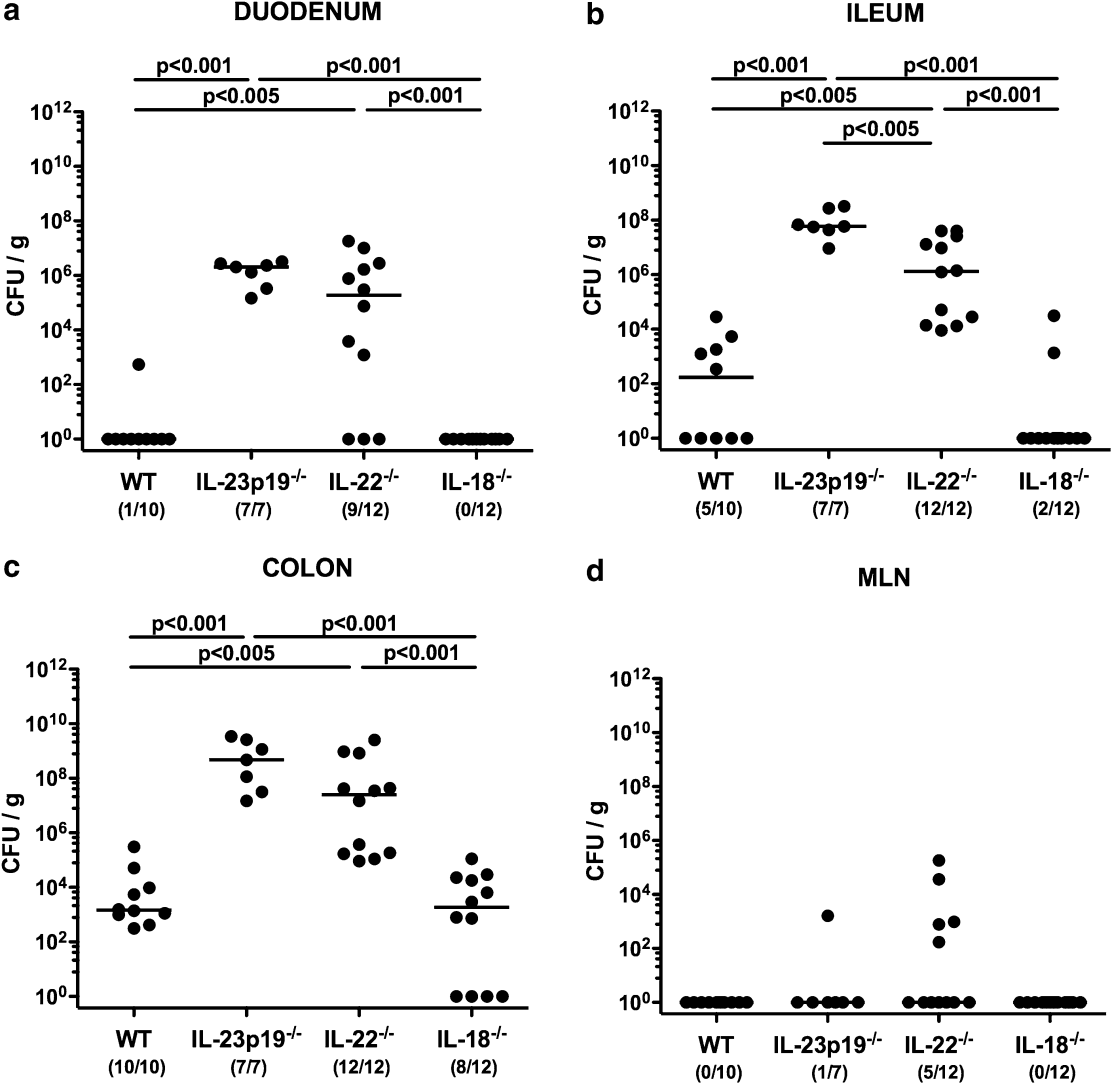
Intestinal *E. coli* loads in *C. jejuni* infected mice lacking IL-23p19, IL-22 or IL-18. Conventional wildtype (WT), IL-23p19^−/−^, IL-22^−/−^ and IL-18^−/−^ mice were perorally infected with *C. jejuni* strain ATCC 43431 by gavage at days 0, 1 and 2. Intestinal commensal *E. coli* loads (*CFU* colony forming units per gram) were determined in luminal samples derived from the **a** duodenum, **b** terminal ileum, **c** colon and homogenates of **d** mesenteric lymph nodes (MLN) at day 14 postinfection by culture. Numbers of mice harboring commensal *E. coli* out of the total number of analyzed animals are given in parentheses. Medians (*black bars*) and levels of significance (p values) determined by Mann–Whitney U test are indicated. Data were pooled from three independent experiments

We next addressed the question whether the difference in susceptibiliy to *C. jejuni* infection observed in gene-deficient and WT mice was due to the respective loads of enterobacteria such as *E. coli* derived from the intestinal commensal microbiota. In fact, naive IL-22^−/−^ mice harbored the highest fecal *E. coli* loads when compared to the remaining groups of mice (p < 0.001; Fig. 3). Interestingly, in IL-18^−/−^ mice lower fecal enterobacterial burdens could be assessed as compared to IL-23p19^−/−^ mice, and furthermore, no enterobacteria at all were isolated in 22.2 % of fecal samples taken from naive IL-18^−/−^ animals by direct plating (Fig. 3). At day of necropsy, *E. coli* loads in duodenum, ileum and colon were higher in IL-23p19^−/−^ and IL-22^−/−^ mice as compared to IL-18^−/−^ and WT animals (p < 0.005–0.001; Fig. 4a–c). Moreover, viable *E. coli* could be cultured from MLNs in 14.3 % of IL-23p19^−/−^ and 41.7 % of IL-22^−/−^ mice (Fig. 4d). As for *C. jejuni* infection, commensal *E. coli* translocated from the intestines to extra-intestinal compartments such as spleen, liver and kindey in single cases only, but could never be detected in cardiac blood (Additional file 2: Fig. S2).

Taken together, IL-22^−/−^ mice that were highly susceptible to stable *C. jejuni* infection displayed higher intestinal enterobacterial concentrations as compared to colonization resistent WT mice. In fecal samples of some IL-18^−/−^ mice that could also be readily colonized by the pathogen, however, commensal enterobacteria could not be detected by direct plating pointing towards additional factors rendering the host susceptible to pathogenic infection.

### Microscopic sequelae of *C. jejuni* infection in conventional mice lacking IL-23p19, IL-22 or IL-18

**Fig. 5:**
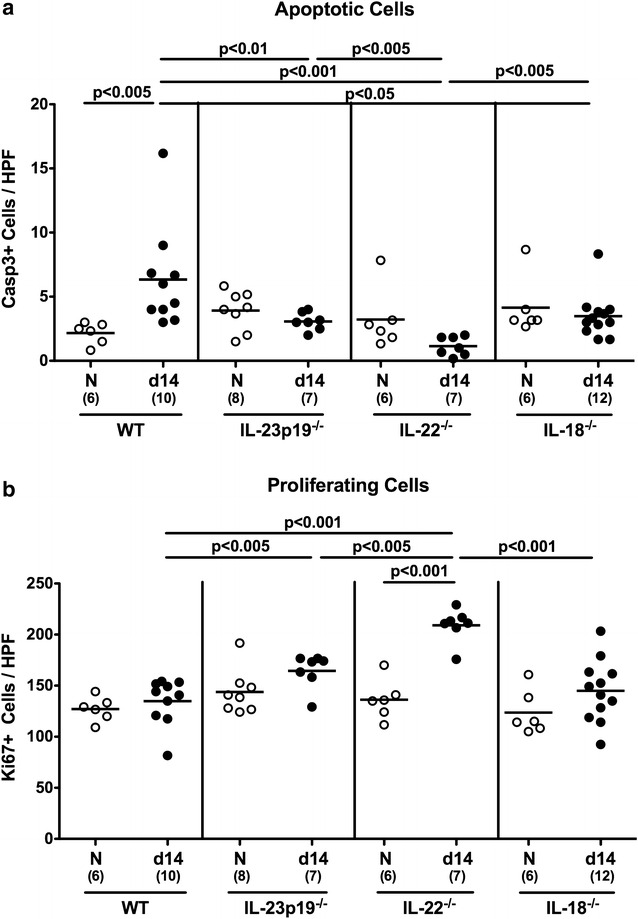
Apoptotis and cell proliferation in the colon of *C. jejuni* infected mice lacking IL-23p19, IL-22 or IL-18. Conventional wildtype (WT), IL-23p19^−/−^, IL-22^−/−^ and IL-18^−/−^ mice were perorally infected with *C. jejuni* strain ATCC 43431 by gavage at days 0, 1 and 2. The average number of colonic epithelial **a** apoptotic cells (positive for caspase-3, Casp3) and **b** proliferating cells (positive for Ki67) from at least six high power fields (HPF, 400× magnification) per animal was determined microscopically in immunohistochemically stained colonic paraffin sections at day (d) 14 (*black circles*) postinfection. Naive (N) mice served as uninfected controls (white circles). Medians (*black bars*), levels of significance (p values) determined by Mann–Whitney U test, and numbers of analyzed animals (in parentheses) are indicated. Data were pooled from three independent experiments

We next determined *C. jejuni* induced histopathology in colonic ex vivo biopsies. To address this, colonic paraffin sections were stained for caspase-3 by in situ immunhistochemistry in order to quantitate apoptosis in colonic epithelium, a well recognized diagnostic marker in the histopathological evaluation and grading of intestinal disease including campylobacteriosis [12]. Following *C. jejuni* infection, apoptotic cell numbers increased in WT, but not in mice lacking IL-23p19, IL-22 or IL-18 (p < 0.005), with lowest colonic epithelial counts in IL-22^−/−^ mice at day 14 p.i. (p < 0.005 vs remaining groups; Fig. 5a). Conversely, IL-22^−/−^, but also IL-23p19^−/−^ mice displayed higher numbers of Ki67-positive cells in colonic epithelial layers as compared to WT mice (p < 0.005; Fig. 5b), indicative for more pronounced cellular proliferation. Hence, despite highest intestinal pathogenic loads, IL-22^−/−^ mice did not exhibit *C. jejuni* induced intestinal apoptosis, but on the contrary, even higher cell proliferating responses as potential measure counteracting eventual cell damage.

### Colonic pro-inflammatory immune responses upon *C. jejuni* infection of conventional mice lacking IL-23p19, IL-22 or IL-18

**Fig. 6:**
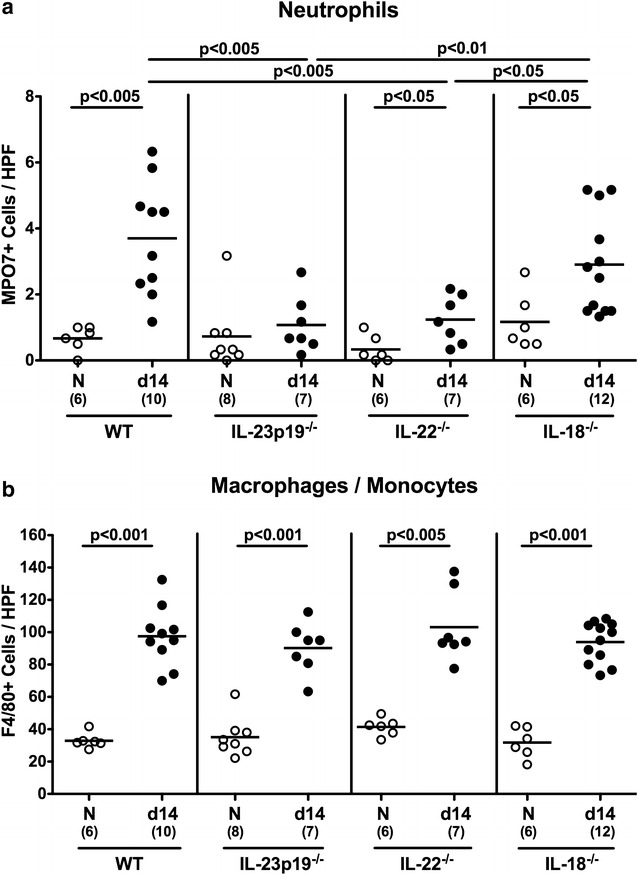
Colonic innate immune cells in *C. jejuni* infected mice lacking IL-23p19, IL-22 or IL-18. Conventional wildtype (WT), IL-23p19^−/−^, IL-22^−/−^ and IL-18^−/−^ mice were perorally infected with *C. jejuni* strain ATCC 43431 by gavage at days 0, 1 and 2. The average number of colonic epithelial cells positive for **a** myeloperoxidase-7 (MPO7; neutrophils) and **b** F4/80 (macrophages and monocytes) from at least six high power fields (HPF, 400× magnification) per animal was determined microscopically in immunohistochemically stained colonic paraffin sections at day (d) 14 (*black circles*) postinfection. Naive (N) mice served as uninfected controls (*white circles*). Medians (*black bars*), levels of significance (p values) determined by Mann–Whitney U test, and numbers of analyzed animals (in parentheses) are indicated. Data were pooled from three independent experiments

Given that recruitment of pro-inflammatory immune cells to sites of inflammation is a hallmark of campylobacteriosis [12], we next surveyed the numbers of distinct innate immune cell populations by in situ immunohistochemical staining of colonic biopsies. Following *C. jejuni* infection, numbers of colonic MPO7-positive neutrophils increased in WT, IL-22^−/−^ and IL-18^−/−^, but not IL-23p19^−/−^ mice (p < 0.05−0.005; Fig. 6a). At day 14 p.i., colonic neutrophil numbers were lower in IL-23p19^−/−^ and IL-22^−/−^ as compared to WT mice (p < 0.005; Fig. 6a). Moreover, numbers of F4/80-positive macrophages and monocytes increased in large intestinal mucosa and lamina propria of infected mice (p < 0.005), but did not differ between genotypes at day 14 p.i. (Figure 6b).

**Fig. 7:**
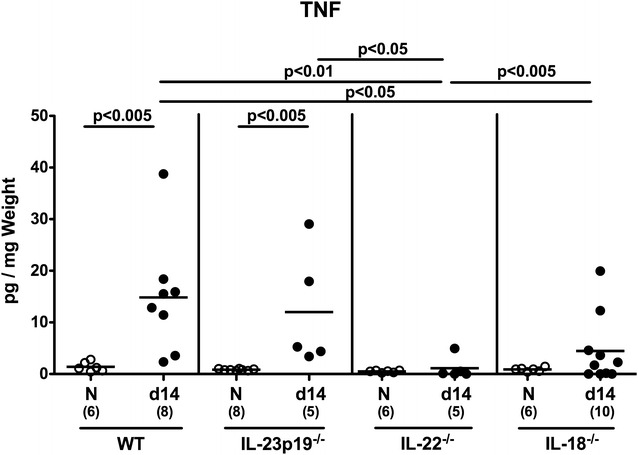
TNF secretion in colonic ex vivo biopsies derived from *C. jejuni* infected mice lacking IL-23p19, IL-22 or IL-18. Conventional wildtype (WT), IL-23p19^−/−^, IL-22^−/−^ and IL-18^−/−^ mice were perorally infected with *C. jejuni* strain ATCC 43431 by gavage at days 0, 1 and 2. TNF concentrations were determined in supernatants of colonic ex vivo biopsies derived at day (d) 14 (*black circles*) postinfection. Naive (N) mice served as uninfected controls (*white circles*). Medians (*black bars*), levels of significance (p values) determined by Mann–Whitney U test, and numbers of analyzed animals (in parentheses) are indicated. Data were pooled from three independent experiments

We next measured secretion of the pro-inflammatory cytokine TNF in colonic ex vivo biopsies following *C. jejuni* infection. WT and IL-23p19^−/−^, but not IL-22^−/−^ and IL-18^−/−^ mice displayed *C. jejuni*-induced inreased colonic TNF concentrations (p < 0.005; Fig. 7). At day 14 p.i., IL-22^−/−^ mice exhibited lower TNF levels in their large intestines as compared to the remaining infected groups (p < 0.05–0.005; Fig. 7).

### Colonic expression on IL-23p19, IL-22 and IL-18 in *C. jejuni* infected conventional mice lacking IL-23p19, IL-22 or IL-18

**Fig. 8:**
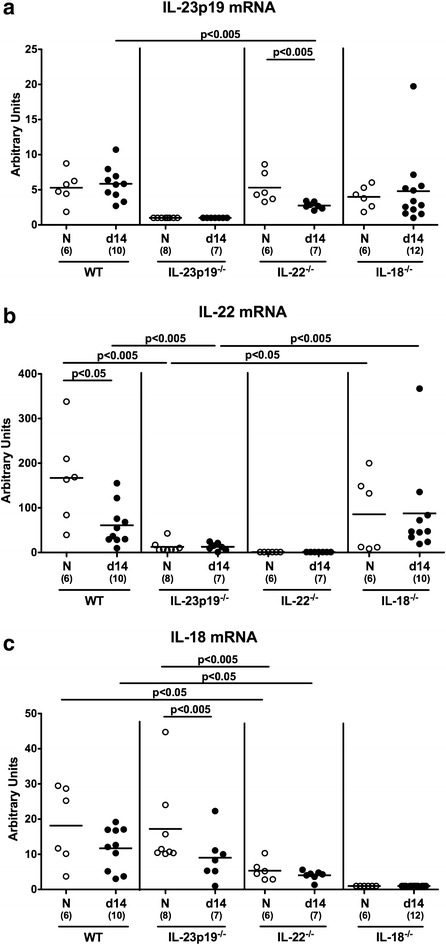
IL-23p19, IL-22, and IL-18 mRNA in colonic ex vivo biopsies derived from *C. jejuni* infected mice lacking IL-23p19, IL-22 or IL-18. Conventional wildtype (WT), IL-23p19^−/−^, IL-22^−/−^ and IL-18^−/−^ mice were perorally infected with *C. jejuni* strain ATCC 43431 by gavage at days 0, 1 and 2. **a** IL-23 p19, **b** IL-22, and **c** IL-18 mRNA expression levels were determined in colonic ex vivo biopsies at day (d) 14 postinfection (*black circles*) by Real Time PCR and expressed in Arbitrary Units (fold expression). Naive (N) mice served as uninfected controls (*white circles*). Medians (*black bars*), levels of significance (p values) determined by Mann–Whitney U test, and numbers of analyzed animals (in parentheses) are indicated. Data were pooled from three independent experiments

We next addressed whether colonic IL-23p19, IL-22 and IL-18 mRNA were differentially expressed in IL-23p19^−/−^, IL-22^−/−^ and IL-18^−/−^ mice upon *C. jejuni* infection. In infected IL-22^−/−^ mice, colonic IL-23p19 was down-regulated and mRNA expression levels lower as compared to infected WT mice (p < 0.005; Fig. 8a). In naive and infected IL-23p19^−/−^, but not IL-18^−/−^ mice, colonic IL-22 mRNA levels were lower as compared to the respective WT controls (p < 0.005; Fig. 8b). In naive and infected IL-22^−/−^, but not IL-23p19^−/−^ mice, however, lower IL-18 mRNA concentrations could be measured as compared to naive and infected WT animals, respectively (p < 0.05; Fig. 8c). Hence, cytokines of the IL-23/IL-22/IL-18 axis appear in fact to be differentially expressed and regulated during *C. jejuni* infection.

## Discussion

Cytokines of the IL-23/IL-22/IL-18 axis are pivotally involved in host defence and in mediating and regulating inflammatory immune responses upon bacterial and parasitic infection [21, 25, 26]. We here investigated whether IL-23, IL-22 and IL-18 were actors in the orchestrated interplay between *C. jejuni*, host microbiota and immune system. Interestingly, conventionally colonized mice lacking IL-23p19, IL-22 or IL-18, but not WT control mice were susceptible to *C. jejuni* infection and could be readily colonized with highest bacterial loads in the terminal ileum and colon. In line with our previous studies, physiological colonization resistance prevented WT mice harboring a conventional intestinal microbiota from pathogenic infection [12, 15]. Modulation of the intestinal microbiota towards elevated luminal commensal enterobacterial (i.e. *E. coli*) loads, however, was a sufficient way to override colonization resistance, given that *C. jejuni* infection was facilitated upon feeding conventional adult mice viable *E. coli* via the drinking water [15] or a Western style diet, for instance [14]. Interestingly, only susceptible IL-22^−/−^, but neither IL-23p19^−/−^, nor IL-18^−/−^ mice harbored elevated commensal intestinal *E. coli* loads when compared to resistant WT mice. This is well in line with a previous report showing an elevated abundance of the phylum Proteobacteria such as commensal *E. coli* in the intestines of IL-22^−/−^ mice [27]. An altered microbiota composition rendered IL-22^−/−^ mice even more susceptible to colitis development than WT mice and can be explained by the fact that IL-22 plays a critical role in regulating the host microbiota composition due to its important antimicrobial properties including induction of antimicrobial peptides such as β-defensins, but also of the mucosal barrier forming mucins [27–30]. Hence, other so far unidentified host-related factors might predispose IL-18^−/−^, but also IL-23p19^−/−^ mice to *C. jejuni* infection.

We have recently shown that 3-weeks-old conventional infant mice develop self-limiting acute enteritis within 1 week following peroral *C. jejuni* infection immediately after weaning [15, 31, 32]. Notably, infant mice also harbored higher intestinal commensal *E. coli* loads in their gastrointestinal tract as compared to adult mice, subsequently facilitating *C. jejuni* infection [15, 31, 32]. As shown by us previously, colonic IL-23p19, IL-22 and IL-18 mRNA were upregulated in *C. jejuni* infected infant mice [22]. Moreover, following peroral infection of conventional adult mice with *Arcobacter butzleri* sharing taxonomic relationships to Campylobacterales, cytokines of the IL-23/IL-22/IL-18 axis were regulated not only in a strain and time course of infection, but also tissue dependent fashion. Whereas in the colon IL-22 and IL-18 were up-regulated upon *A. butzleri* infection, IL-23p19 and IL-22 mRNA levels increased in the small intestines of infected conventional adult WT mice [33, 34].

In our present study, despite stable *C. jejuni* infection, mice lacking IL-23p19, IL-22 or IL-18 exhibited even lower colonic epithelial apoptotic cell numbers as compared to WT mice at day 14 p.i., whereas higher numbers of proliferating cells could be observed in the colonic epithelium of infected IL-22^−/−^ mice, thereby counteracting potential *C. jejuni* induced cell death. These results are in part supported by our recent study in infant mice that were infected with a different *C. jejuni* strain (namely 81–176): 2 weeks following peroral infection, less pronounced colonic apoptosis and conversely, more distinct proliferative measures could be observed in the large intestines of infant IL-22^−/−^, but also IL-18^−/−^ as compared to WT mice [35]. In fact it is somewhat surprising that even though the pathogen was expelled from the intestinal tract of infected infant WT mice here, increased numbers of colonic epithelial apoptotic cells could be observed. This observation, however, is well in line with results from our previous studies in different infection models [22, 35, 36]. We hypothesize that the pathogen does not necessarily need to be permanently abundant in the intestinal tract to evoke (early) host responses with subsequent pro-inflammatory sequelae and tissue damage including intestinal apoptosis. Hence, it is rather the initial hit of infection that tips the balance towards immunopathological responses and potential counter-regulatory (i.e. proliferative) measures postinfection [22, 35, 36].

Here, less distinct colonic epithelial apoptosis were accompanied by lower abundance of neutrophilic granulocytes within the large intestinal mucosa and lamina propria of infected IL-23p19^−/−^ and IL-22^−/−^ as compared to WT control mice, and paralleled by lower colonic TNF secretion in IL-22^−/−^ and IL-18^−/−^ mice than in WT animals, which also held true during the early phase (i.e. day 6) of *C. jejuni* infection of infant mice [35].

The cytokines of the IL-23/IL-22/IL-18 axis appear in fact to be differentially expressed and regulated during murine *C. jejuni* infection. Our present study revealed that (1) colonic IL-23p19 mRNA expression was lower in infected IL-22^−/−^ mice than WT mice (2) and vice versa, i.e. IL-22 mRNA was lower in IL-23p19^−/−^ vs. WT mice at day 14 p.i., whereas (3) IL-18 mRNA was down-regulated in large intestines of naive and infected IL-22^−/−^ mice. These data are in part supported by results derived from the infant mouse infection model, given that in naive as well as in *C. jejuni* infected infant IL-22^−/−^ mice, colonic IL-23p19 and IL-18 mRNA were down-regulated, which also held true for IL-18 mRNA expression levels in naive and infected infant IL-23p19^−/−^ as compared to WT control mice [36]. It is most likely that the observed differences in expression data derived from infection studies with infant versus adult mice are due to age-dependent differences in host-factors including intestinal microbiota compositon and subsequently the intraluminal milieu as well as the maturity of immune cell subsets and cells producing antimicrobial peptides for instance. Information about the distinct regulatory pathways within the IL-23/IL-22/IL-18 axis following bacterial (i.e. *C. jejuni*) infection are very limited. Whereas IL-23 was highlighted as a key regulator of mucosal immune responses following intestinal infection and inflammation [37] including *T. gondii* induced ileitis [20], IL-22 was shown to be effective in antimicrobial defense directed against *C. jejuni* [28]. Moreover, in human intestinal ex vivo biopsies IL-22 was upregulated upon *C. jejuni* infection [38], whereas elevated IL-22 concentrations were observed in the intestines of *C. jejuni* infected IL-10^−/−^ mice [17]. Data regarding the role of IL-18 in *C. jejuni*-host interaction, however, are scarce. *C. jejuni* infection of three different cell lines derived from pre-malignant Barret’s esophagus was accompanied by an up-regulation of IL-18 gene expression [39]. Following infection of differentiated THP-1 macrophages with an adherent and invasive *C. concisus* strain, genes encoding IL-23 and IL-18, but not IL-22, were regulated as assessed by transcriptomic and proteomic analyses [40]. Our previous *A. butzleri* infection studies in gnotobiotic IL-10^−/−^ mice further revealed that in the colon IL-18 mRNA levels were elevated during both the early and late phase of infection, whereas colonic IL-22 mRNA was upregulated during the former only [33, 34].

In conclusion, the regulatory pathways within the IL-23/IL-22/IL-18 axis following *C. jejuni* infection need to be further unraveled in future studies in order to improve our understanding of the distinct molecular mechanisms underlying campylobacteriosis.

## Additional files

10.1186/s13099-016-0106-4 Extraintestinal translocation of viable intestinal *C. jejuni* strain 81–176 in perorally infected mice lacking IL-23p19, IL-22 or IL-18. Conventional wildtype (WT), IL-23p19^−/−^, IL-22^−/−^ and IL-18^−/−^ mice were perorally infected with *C. jejuni* strain 81–176 by gavage at day 0 and day 1. Pathogenic translocation to extraintestinal compartments was assessed by determining *C. jejuni* strain 81–176 loads (CFU, colony forming units per gram) in (A) spleen (B) liver (C) kidney, and (D) cardiac blood at day 14 postinfection by culture. Numbers of mice harboring the pathogen out of the total number of analyzed animals are given in parentheses.
10.1186/s13099-016-0106-4 Extraintestinal translocation of viable intestinal commensal *E. coli* in perorally *C. jejuni* strain 81-176 infected mice lacking IL-23p19, IL-22 or IL-18. Conventional wildtype (WT), IL-23p19^−/−^, IL-22^−/−^ and IL-18^−/−^ mice were perorally infected with *C. jejuni* strain 81-176 by gavage at day 0 and day 1. Intestinal translocation of viable *E. coli* derived from the commensal intestinal microbiota to extraintestinal compartments was assessed by determining bacterial loads (CFU, colony forming units per gram) in (A) spleen (B**)** liver (C**)** kidney, and (D) cardiac blood at day 14 postinfection by culture. Numbers of mice harboring *E. coli* out of the total number of analyzed animals are given in parentheses, and medians (black bars) are indicated. Data were pooled from three independent experiments.

## Abbreviations

ILCs: innate lymphoid cells
Th: T helper
WT: wildtype
PCR: polymerase chain reaction
CFU: colony forming units
PBS: phosphate buffered saline
p.i.: post infection
MLNs: mesenteric lymph nodes
HPRT: hypoxanthine-phosphoribosyltransferase
