# Supplementary figures and images for: The IL-23/IL-22/IL-18 axis in murine Campylobacter jejuni infection
Source: Gut Pathog. 2016 Jul 6;8:21. doi: 10.1186/s13099-016-0106-4 (PMC4934010; doi:10.1186/s13099-016-0106-4)

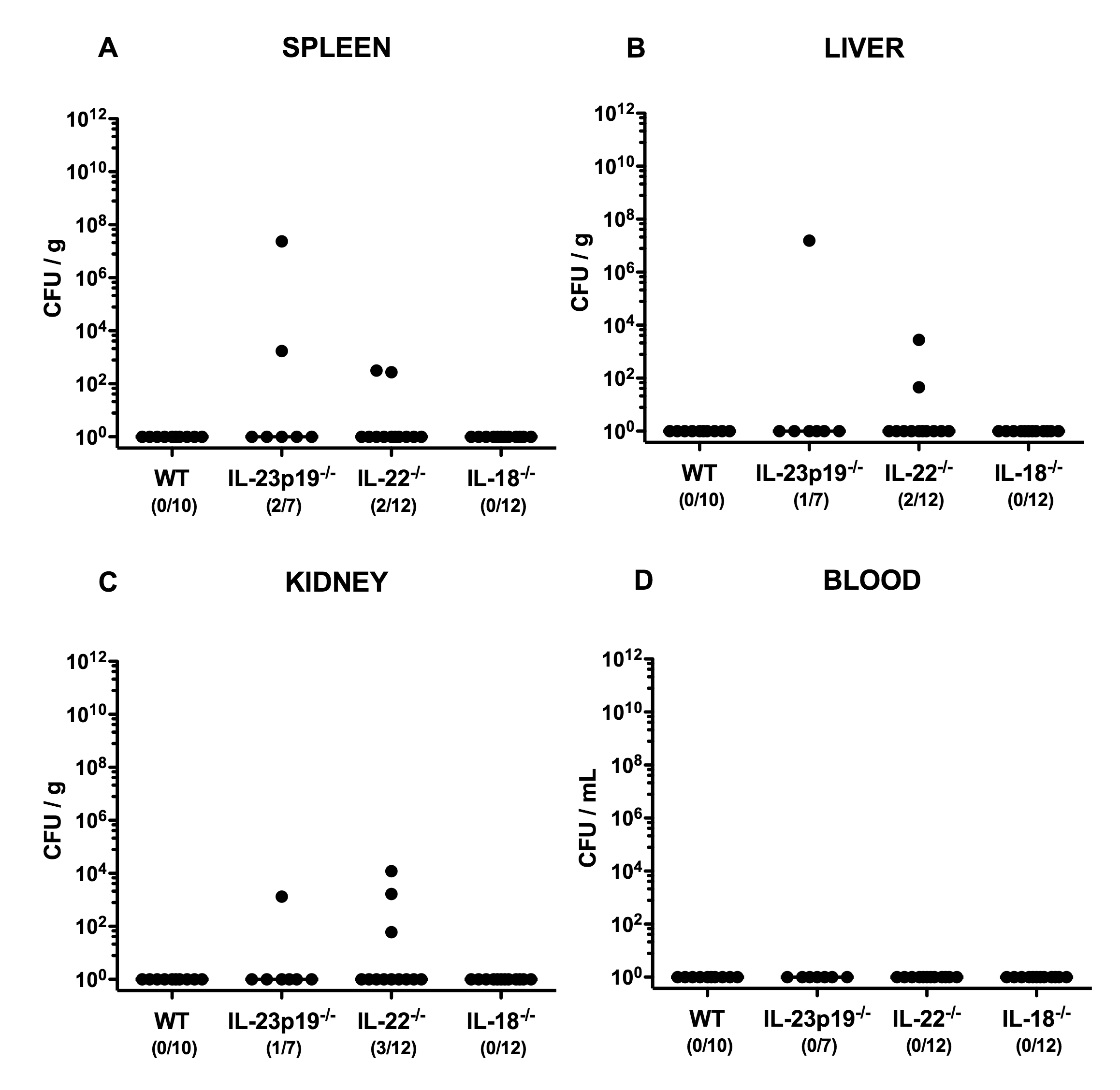

Supplement: Supplementary file 1 — 10.1186/s13099-016-0106-4 Extraintestinal translocation of viable intestinal C. jejuni strain 81–176 in perorally infected mice lacking IL-23p19, IL-22 or IL-18. Conventional wildtype (WT), IL-23p19−/−, IL-22−/− and IL-18−/− mice were perorally infected with C. jejuni strain 81–176 by gavage at day 0 and day 1. Pathogenic translocation to extraintestinal compartments was assessed by determining C. jejuni strain 81–176 loads (CFU, colony forming units per gram) in (A) spleen (B) liver (C) kidney, and (D) cardiac blood at day 14 postinfection by culture. Numbers of mice harboring the pathogen out of the total number of analyzed animals are given in parentheses. [file 13099_2016_106_MOESM1_ESM.tiff]

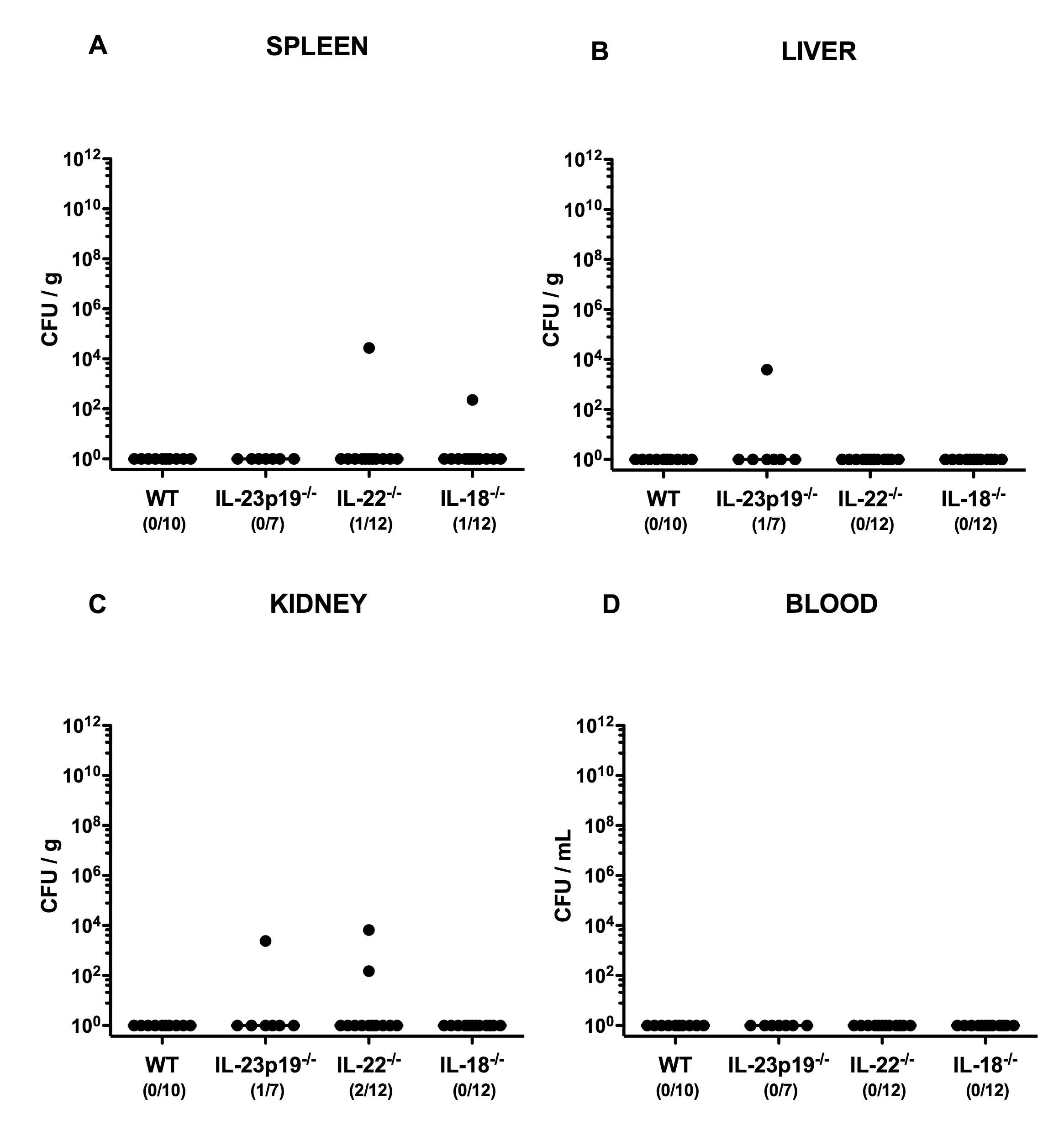

Supplement: Supplementary file 2 — 10.1186/s13099-016-0106-4 Extraintestinal translocation of viable intestinal commensal E. coli in perorally C. jejuni strain 81-176 infected mice lacking IL-23p19, IL-22 or IL-18. Conventional wildtype (WT), IL-23p19−/−, IL-22−/− and IL-18−/− mice were perorally infected with C. jejuni strain 81-176 by gavage at day 0 and day 1. Intestinal translocation of viable E. coli derived from the commensal intestinal microbiota to extraintestinal compartments was assessed by determining bacterial loads (CFU, colony forming units per gram) in (A) spleen (B) liver (C) kidney, and (D) cardiac blood at day 14 postinfection by culture. Numbers of mice harboring E. coli out of the total number of analyzed animals are given in parentheses, and medians (black bars) are indicated. Data were pooled from three independent experiments. [file 13099_2016_106_MOESM2_ESM.tiff]
